# In Vitro Growth of Human Follicles: Current and Future Perspectives

**DOI:** 10.3390/ijms25031510

**Published:** 2024-01-26

**Authors:** Clara Malo, Sara Oliván, Ignacio Ochoa, Ariella Shikanov

**Affiliations:** 1 Tissue Microenvironment (TME) Lab, Aragón Institute of Engineering Research (I3A), University of Zaragoza, 50018 Zaragoza, Spain; soligar@unizar.es (S.O.); iochgar@unizar.es (I.O.); 2 Institute for Health Research Aragón (IIS Aragón), 50009 Zaragoza, Spain; 3 Centro de Investigación Biomédica en Red de Bioingeniería, Biomateriales y Nanomedicina (CIBER-BBN), Instituto de Salud Carlos III, 50018 Zaragoza, Spain; 4 Department of Biomedical Engineering, University of Michigan, Ann Arbor, MI 48109, USA; shikanov@umich.edu; 5 Department of Obstetrics and Gynecology, University of Michigan, Ann Arbor, MI 48109, USA; 6 Cellular and Molecular Biology Program, University of Michigan, Ann Arbor, MI 48109, USA

**Keywords:** cancer, cryopreservation, fertility preservation, in vitro follicular growth, ovarian tissue, organ-on-chip, prepubertal, three-dimensional culture systems, two-dimensional culture systems

## Abstract

Ovarian tissue cryopreservation is gaining importance as a successful method to restore fertility to girls and young women at high risk of sterility. However, there are concerns regarding the safety of transplantation after ovarian tissue cryopreservation due to the high risk of reintroducing cancer cells and causing disease recurrence. In these cases, the development of culture systems that support oocyte development from the primordial follicle stage is required. Notable achievements have been reached in human follicle in vitro growth in the past decade. Currently, systems for the in vitro culture of ovarian tissue are based on two-dimensional substrates that do not support the survival of follicles or recapitulate the mechanical heterogenicity in the mammalian ovary. Recognition of the importance of special arrangements between cells has spurred research in three-dimensional culture systems, and the provision of a precise culture system that maximizes the diffusion of nutrients and gases through the follicles has raised interest in advanced biomimetic models. The current review critically examines various culture systems employed for the in vitro development of follicles, with a particular focus on solutions utilizing Organ-on-a-Chip (OOC) technology. The emphasis on OOC technology underscores its role as a promising avenue in ensuring the successful cultivation and maintenance of follicular structures during the culture period.

## 1. Introduction

Cancer is the leading cause of death in children around the world. Yearly, approximately 87,000 girls aged from 0 to 19 years old are diagnosed with cancer worldwide, according to the World Health Organization. Advances in childhood cancer research have increased the cure rate to over 80% in high-income countries [1]. However, the therapy side-effects on patient reproductive health have received little attention, with fertility failure being one of the most detrimental consequences. The risk of gonadotoxicity in oncologic patients after treatment is up to 80% depending on the type of anticancer treatment [2].

**Figure 1:**
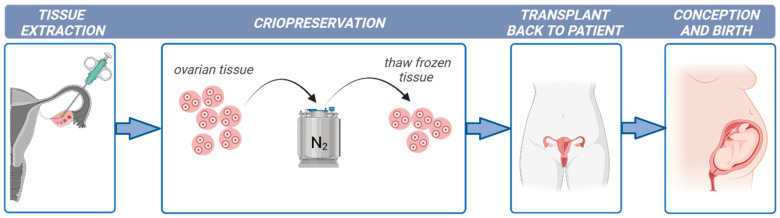
Fertility preservation protocol in young women suffering from cancer. Created with BioRender.com.

While fertility, in principle, can be preserved by freezing oocytes or embryos in post-pubertal women, the primary fertility preservation option that exists for pre-pubertal girls or oncologic patients undergoing immediate gonadotoxic cancer treatment is ovarian tissue cryopreservation (OTC) and its subsequent transplantation (OTT) [3,4] (illustrated in Figure 1). In addition, other non-oncological diseases such as autoimmune and hematological disorders are treated with chemotherapy and radiotherapy and require fertility preservation procedures [5].

Currently, it is estimated that more than 10,000 girls and women worldwide have undergone OTC [6], and more than 200 children have been conceived from OTT [7]. A well-documented series of OTTs from five European centers has shown that the chance of having one or more live births after a transplant is 26% [8]. Another multicenter study even reported a 41.6% chance of at least one delivery after OTT [9]. However, OTT is an invasive surgical procedure and carries the risk of reintroducing malignant cells present in the ovarian cortex retrieved from patients with blood-borne cancers. Data have demonstrated that for most cancers, the risk is likely to be low, and the most significant risk is associated with hematological malignancies, particularly leukemia, which is also most prevalent in children. Thus, OTT is not advised in these cases. For these patients, the in vitro growth (IVG) of follicles could be a promising alternative to transplantation.

There are still several challenges to overcome before IVG can be applied as an approach for yielding competent oocytes. This review aims to summarize the current status of isolated follicles’ culture to produce oocytes for fertility preservation, mainly indicated for young women suffering from cancer.

## 2. Folliculogenesis in Human Ovaries

The normal development of the human ovary starts from day 26 of pregnancy when human primordial germ cells arrive from the yolk sac to the gonad (oogonia) [10]. Then, after many mitotic division cycles of the oogonia, meiotic division starts and the diplotene stage is achieved, when the oogonia become the larger oocyte. Follicle formation in humans begins in the fourth month, when a single layer enclosed in flat membrane cells called granulosa cells (GCs) surrounds the oocyte, forming the primordial follicles (30–50 µm in diameter) [11]. At birth, the female germline reserve of primordial follicles is located in the rigid cortex and contains oocytes arrested in the diplotene stage of the prophase of the first meiotic division. Primordial follicles are recruited throughout life to enter folliculogenesis and expand from the cortex towards the medulla, reaching antral follicles stages.

**Figure 2:**
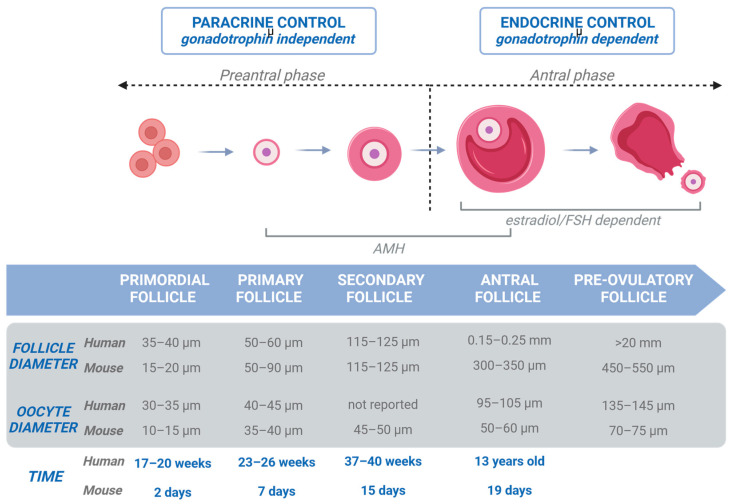
In vivo folliculogenesis. Folliculogenesis can be separated into early folliculogenesis and late folliculogenesis. Early folliculogenesis is under paracrine control and is gonadotrophin-independent, whereas late folliculogenesis is under endocrine control and is gonadotrophin-dependent. Follicle and oocyte promedium sizes for human and mouse are mentioned, as well as time of appearance [13]. Created with BioRender.com.

Follicle development involves a series of precisely regulated biological events (illustrated in Figure 2): activation of primordial follicles (35–40 μm) with GCs becoming cuboidal (called primary follicles; 50–60 μm diameter), growth of the primary follicles with an increase in the proliferation rate of GCs yielding a multilaminar granulosa layer (called secondary follicles; 115–125 μm diameter), creation of a theca layer that will produce steroid hormones through a complex interaction with the GCs, development of an antral cavity, and rupture of the ovulatory follicle releasing a cumulus–oocyte complex (COC) after antral follicles reach 20 mm in diameter [11,12,13].

Oocytes arrested in the meiotic stage of prophase I in early-stage follicles must acquire the developmental competence necessary to resume meiosis and complete maturation as well as support fertilization and embryonic growth. Russell et al. [14] demonstrated the crucial role of bidirectional communication between the oocyte and GCs in obtaining competent oocytes and future embryos because of secreted paracrine factors that promote the growth of both cells. The oocyte is unable to transport several amino acids or carry out glycolysis and cholesterol biosynthesis without the cooperation of granulosa cells [15].

The ovarian tissue in all young females that is harvested for cryopreservation contains primordial follicles. At birth, the human ovary contains about 1–2 million primordial follicles. By the time a girl enters puberty, only about 25% (300,000 oocytes) remain. However, the majority of oocytes from these primordial follicles never ovulate and therefore never contribute to reproduction [16]. The ability to rescue and grow these follicles in vitro to a mature stage with a competent oocyte would significantly improve fertility preservation and restoration options.

## 3. Culture Systems

Cells and tissue require an optimized in vitro culture system that closely mimics their natural conditions. In vitro culture provides nutrients and gases to tissue for follicular development and growth. The development of an in vitro system that supports maturing primordial follicles to matured stages would revolutionize the fertility preservation options for this young population, harnessing the full reproductive potential of the tissue and avoiding the degeneration of the majority of the primordial follicles recruited in the growing pool.

Studies on the in vitro culture of ovarian fragments or isolated follicles were largely used as a research tool to investigate folliculogenesis, a complex process that is far from being fully understood in all mammal species [17,18,19]. Now, they also represent a potential alternative to restore fertility. However, complete folliculogenesis from primordial stages to the birth of live offspring has only been achieved in mice [20]. Several approaches have been performed to develop systems supporting the in vitro development of primordial follicles in humans. Currently, most of the incubations are based on static systems, with the manual exchange of medium every 24 to 72 h. Conventional in vitro systems that include two-dimensional (2D) and three-dimensional (3D) cultures have serious pitfalls that make them unstable for growing follicles in vitro. The recently developed OOC technology provides new in vitro models to serve as tissue proxies to bridge the gap between in vitro and in vivo in many organs but is still in its infancy for its application to ovarian tissue.

### 3.1. Two-Dimensional Culture Systems

**Figure 3:**
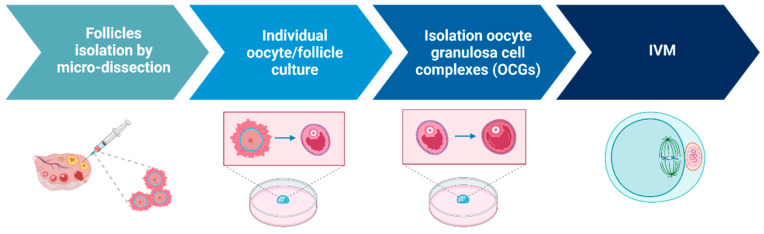
Diagrammatic representation of a multi-step culture system to support IVG of oocytes from human primordial follicles through to maturation as described by McLaughlin et al. [28]. Created with BioRender.com.

The first attempts for IVG in mammals were conducted using 2D culture systems [21,22]. Two-dimensional systems used for in vitro follicle culture include multi-wells [23], microdrops [24], or membranes coated with extracellular proteins [25,26]. Only in mice has the production of live young from cultured primordial follicles been successful [20,21]. This research group developed a two-stage culture system: primordial follicles were grown in tissue to secondary follicles, followed by isolation of the secondary follicles and culture to mature oocytes. However, only 59 live offspring (5.7% of embryos transferred) were obtained [20]. Achieving live birth from in vitro-cultured primordial follicles in mice is an important step in understanding some of the universal mammalian mechanisms of folliculogenesis, but this approach has not been translated to humans [27], mainly due to the longer duration of follicle development (10–12 days in mice compared to 2–3 months in humans) and the larger sizes reached by the follicles in humans (0.5–0.6 mm in mice compared to 6 mm in humans). There is so far only one paper reporting successful oocyte meiotic maturation from primordial follicles from human adult ovaries grown using a multi-step culture strategy (in vitro activation (IVA) of primordial follicles, isolation of secondary follicles, isolation of cumulus oocytes complex (COCs), in vitro maturation (IVM) of oocytes) and a static system (steps illustrated in Figure 3). The presence of the Metaphase II spindle was confirmed in these IVG oocytes but further information about their fertilization and developmental potential is required to determine if they can be a source to preserve fertility [28].

The major challenge of follicular IVG is to ensure the growth of the primary follicle, the development of granulosa and theca cells, and subsequently the development of an antrum [29]. Probably the most critical limitation is the failure to maintain the follicular spherical structure, disrupting the cellular interactions between the oocyte and GCs and compromising the further in vitro development. Several of the gap junctions and the intercellular communication between the oocyte and GCs are weakened during in vitro culture [30]. The conventional 2D culture of follicles impedes spherical growth and the preservation of the special arrangements between GCs and the oocyte tend to decrease, leaving the oocyte denuded and unable to complete the maturation process. In longer culture periods as when culturing primordial follicles, the importance of the special arrangements is even greater, leading to an interest in 3D culture to recapitulate the structure and function of the follicles [31].

### 3.2. Three-Dimensional Culture Systems

During the physiological reproductive cycle process, the ovarian microenvironment is in constant remodeling, providing clues to the potential role of these microenvironmental aspects in this process. Maintenance of the intricate 3D architecture and GCs and oocyte cell interaction may be critical for the successful in vitro maturation of follicles. Respecting its 3D structure is therefore crucial to maintain proper follicular physiology and obtain responses resembling the expected behavior of follicles in vivo. The 3D culture involves the use of the homogeneous hanging drop or hydrogel encapsulation to preserve the architecture. The early antral follicles culture is strongly influenced by the composition and architecture of its supporting tissue. This generates the need to develop extracellular matrixes and biomaterials that could imitate the ovarian physiologic milieu for optimal follicle development [32,33].

In addition to the spatial arrangement of the cells, the extracellular matrix (ECM) is a structural support network made up collagen, laminin, and fibrinogen, and is increasingly recognized as a master regulator in the communication between cells and in cell differentiation [34]. Matrices are needed to support follicle growth and maturation in 3D culture systems. Several biomaterials such as collagen, alginate, or Matrigel have been explored as an alternative to mimic the ECM within the ovary and to encapsulate and support human secondary and antral follicles. Although some attempts have been successful for culturing human follicles, collagen presents a few challenges such as a limited transparency for monitoring follicular development or the accelerated shrinkage of this material during prolonged culture. One commercially available ECM tested for follicular growth is Matrigel [35,36]. Matrigel is composed of collagen IV, laminin, fibronectin, entactin, and a variety of factors. Hovatta et al. [37] demonstrated a higher survival of follicles in frozen–thawed human ovarian tissue placed on Matrigel. There have been promising results with alginate hydrogel [38], currently the most widely applied biomaterial. The alginate has been applied to culture COCs [39,40,41,42,43]. Promising results have been obtained in humans with the production of meiotically competent oocytes after the in vitro maturation of isolated follicles in calcium alginate hydrogel [44]. Although human follicles isolated from cryopreserved ovarian tissue and then cultivated in calcium alginate hydrogel reached a survival rate of 90%, more studies are needed to verify that this system can maintain its morphology and its functionality can be maintained in this matrix [45].

**Figure 4:**
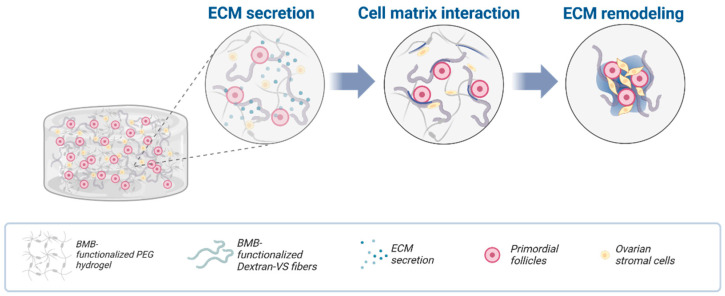
Diagram illustrating the encapsulation of follicles and cells within degradable PEG hydrogels containing DexVS fibers and functionalized with ECM-sequestering BMB peptides. In the presence of BMB-functionalized Dextran-VS fibers, extracellular matrix (ECM) secreted by cells, including laminin and collagen, is sequestered along DexVS fibers. This facilitates cell adhesion and the organization of cells into tissue-like aggregates, which subsequently undergo restoration. Adapted from Nason-Tomaszewski et al. [52].

Other scaffold materials have been reported for IVG such as polyethylene glycol hydrogels [46,47,48], agarose [49,50], or hyaluronic acid [51]. Nason-Tomaszewski et al. [52] used a fully synthetic hydrogel composite material to control the templating of a cell-secreted ECM and allow the aggregation and assembly of follicular organoid-like structures for a long-term culture (Figure 4).

### 3.3. Critical Cell–Cell and Cell–Matrix Interactions for Improved Oocyte Survival and Growth

Nonetheless, 3D culture still presents numerous challenges. For instance, the role of mechanical signaling has been mostly overlooked. There is increasing evidence that physical properties of the ECM play a critical role in follicle development. Indeed, mechanical stiffness may impact cellular proliferation and differentiation and even oocyte-specific gene expression levels in oocytes [53]. For instance, Heise et al. [54] found that the microencapsulated follicles stimulated with follicle-stimulated hormones (FSHs) did not reach the size observed in unencapsulated follicles. Clearly, the mechanical stiffness gradient in the ovary has an impact on the follicular growth but there are other physical properties (stiffness, compositions, porosity) of the matrix that need to be taken into account. For example, primordial follicles survive and maintain follicular integrity when cultured in a rigid environment (2% alginate), but as they expand to the medulla, they require softer environments (0.25% alginate) [55]. To mimic this heterogenicity in the mammalian ovary, Tomaszewski et al. [48] used a functionalized degradable poly(ethylene glycol) hydrogel to recapitulate the native ECM composition, improving the maturation rate of the oocytes in IVG.

Combining different imaging modalities, Ouni et al. [56] studied some biophysical characteristics of the ovary microenvironment at different stages of a woman’s reproductive life, concluding that there is a correlation between rigidity and fertility. This link between ECM stiffness and antrum formation was also demonstrated in a study in rodents with the greatest antrum formation observed at 0.7% alginate hydrogel compared to 1.5 and 3% [40]. In contrast, an opposite link between rigidity and follicle growth was observed in primates, where follicle survival was higher in 0.5 vs. 0.25% calcium alginate [41]. The ovarian stroma of primates is more rigid than that of rodents, which may benefit from stiffer biomaterials. The physical attributes of the 3D matrix selected for IVG need to be tailored to meet species-specific requirements.

Another important attribute of the biomaterial is the porosity to ensure adequate gas exchange and diffusion of the nutrients. The study by Xu et al. [39] illustrated the opposing influences of the rigidity of the biomaterial at high concentrations and the interference with diffusion and optimal growth.

Within the ovary, there is an increase in vascularization as we move further away from the ovarian cortex up the medulla where the secondary follicles are, suggesting a strong need of oxygenation during the final follicular stages. The effect of oxygen tension on follicle and oocyte development has received little attention. Follicles have traditionally been grown in standard incubators with an atmospheric oxygen concentration. However, the oxygen pressure in the peritoneal cavity where the ovaries are located is approximately 5% O_2_ [57]. Primordial follicles exist in the relatively poorly vascularized cortex of the ovary and an abundance of blood vessels is found in the region of the ovary that contains secondary and antral follicles. It is possible that there is a dynamic oxygen transition from relative hypoxia in primordial follicles to a greater oxygen tension in preantral follicles. A lower oxygen tension during IVM improved blastocyst formation using mouse oocytes [58]. Similar results were observed by Xu et al. [38] when culturing secondary follicles. However, some authors defend the benefits of high oxygen levels in follicular culture [59]. Oxidative stress has been observed when culturing in high oxygen levels during in vitro culture [60]. The need for oxygenation may also require an active perfusion system when dealing with longer culture intervals as demonstrated in oocyte IVM [61]. The difficulty in achieving in vitro oocyte maturation may be due to the dysregulation of follicle development by the exposure of follicles to inappropriate oxygen concentrations. One of the key limitations of the in vitro culture of preantral follicles is oxidative stress by the accumulation of reactive oxygen species (ROS), which can impair follicular development and oocyte quality. Antioxidant supplementation can minimize or eliminate the damage caused by ROS. Limited information about the use of exogenous antioxidants has been reported [62,63,64,65]. The addition of ascorbic acid significantly increased the survival of primary follicles encapsulated in hydrogels but also resulted in the stimulation of cellular contact formation and ECM remodeling [66]. Overall, additional experiments are needed to elucidate the effects of oxygen on the different maturation stages of human follicles, and the combination with microfluidic systems could ensure the right percentage of oxygen at any follicular stage. Other characteristics of the biomaterials such as toxicity, viscosity, the ability to be molded during follicle harvest, and biological usefulness are barely described in the bibliography. This information is essential in order to select the right biomaterial for IVG, tailored towards the different follicle stages being cultured. The shear elastic modulus and diffusion characteristics of the biomaterial must be carefully balanced.

Despite the potential advantages of 3D culture, there is still a controversy about how to achieve an efficient culture system for ovarian tissue. In humans, in vitro follicular maturation can take longer than 120 days with early antral follicles of large diameters, so active perfusion systems may be necessary to assure sufficient nutrient supply to grow multilayer follicles. In a recent review, Dr. Evelyn Tefler [27] explained the importance of developing a 3D dynamic culture system with an emphasis on tissue engineering solutions for maintaining the follicular unit during the culture intervals.

## 4. Future Improvements: Organ-on-a-Chip Technology

Although the activation of growth of primordial follicles has been achieved, a limited number of follicles progress to secondary follicles [67]. Decent results were obtained in primates using expandable matrixes in 3D systems but the fertilization ability of the oocytes obtained was low and no blastocyst development was observed [68,69]. One of the reasons for the limited success of this technology can be found in the lack of more appropriate and physiological culture systems because it does not recapitulate the heterogeneous nature of the ECM in the ovary, with the medulla being much softer than the cortex. The ECM is believed to not only provide a 3D network to support the ovarian tissue architecture but also to regulate (together with hormones and nutrients) cell-ECM and cell–cell interactions that are important for follicle development. Choi et al. [70] revealed the crucial role of mechanical heterogeneity in the ovary in regulating follicle development by producing ovarian microtissues by encapsulating early secondary preantral follicles in microcapsules consisting of a softer, biodegradable collagen (0.5%) hydrogel core and a harder, slowly degradable alginate (2%) hydrogel shell. Folliculogenesis mainly depends upon hormones and nutrients, and their disturbance can cause abnormal follicle growth. Hence, a precise culture system that ensures the diffusion of nutrients and gases within the tissue, maximizing the retention of essential growth factors of oocyte maturation, is needed.

Currently, most of the incubations are based on a static culture system with an annual exchange of energy every 24 to 72 h [41,43,71,72]. Tissue culture can benefit from dynamic systems like micro- or nano-fluidics technologies, allowing the configuration of a system to integrate specific steps of the process in accordance to the needs of the cells.

Furthermore, within the ovary, there is an increase in vascularization as one moves deeper into the medulla where secondary and antral follicles grow, suggesting a stronger need for nutrient diffusion during the last stages of maturation. Human ovarian cortex fragments cultured under flow show improved follicle survival and growth [73]. These results are still preliminary as the number of samples were low and they did not use any kind of matrix to maintain the 3D structure.

Considering the aforementioned aspects, a pioneering technology known as Organ-on-a-Chip (OOC) offers the means to replicate tissue architecture and emulate fluidic conditions in an in vitro setting. Broadly, these systems facilitate the miniaturization of experimental models, resulting in several advantages such as reduced working volumes, quicker reaction times, cost-effectiveness, and enhanced precision and control over experimental designs. This innovative approach holds significant promise in advancing research capabilities and improving the efficiency of experimental processes within the field.

OOC platforms, which are founded on microfluidic devices, facilitate three-dimensional organized cell culture by employing various types of ECMs. This methodology aims to emulate tissue architecture and replicate the cellular environment, mechanisms, and physiological responses of organs in an in vitro setting. The microenvironment is established through dynamic interactions among cells, fluids, and the ECM, influencing cellular processes and functions via biophysical and biochemical signals. A distinctive advantage of OOC lies in its ability to operate under flow conditions, enabling the continual replenishment of media, a process that mirrors in vivo blood supply or interstitial flow. Consequently, fluid flow ensures a consistent and adjustable supply of nutrients and oxygen, along with the supplementation of stage-specific growth factors. This dynamic environment sustains physical interactions while preventing cellular stress induced by the formation and accumulation of reactive oxygen substances [74,75].

Another pertinent aspect of this technology is the capability to tailor the devices based on design specifications including shaped microchannels, compartments, and reservoirs or the materials used to make the devices such as polydimethylsiloxane (PDMS; most prevalent), thermoplastics, or glass. Careful consideration is essential in material selection, as the inherent physicochemical properties of certain materials may impose limitations, including transparency, cost, flexibility or rigidity, and gas permeability, as well as considerations about time-consuming fabrication processes. The choice of material is contingent upon factors such as the intended application of the device, volume requirements, and production costs. Accordingly, the microfabrication methods employed may vary, encompassing techniques such as molding, three-dimensional printing, nanoimprinting lithography, and/or etching. This adaptability in design and material selection allows for a tailored approach that aligns with specific research or application needs [76].

The application of microfluidics to the field of reproduction has gained much attention (sex sorting, fertilization, and embryo culture) [77]. In a recent review, Bodke and Budette [78] discussed the use of microfluidic systems to study the female reproductive tract as these systems recapitulate the multicellular structure and its uses to studying the effects of endocrine-disrupting chemicals and diseases such as ovarian cancer, preeclampsia, and polycystic ovaries. Stejskalová et al. [79], Young and Huh [80], and Zubizarreta and Xiao [81] reviewed recent advances that use bioengineering methods to study female reproduction, including the bioengineering models of the ovary, fallopian tube, uterus, embryo implantation, placenta, and reproductive disease. Recently, there has been increasing interest in utilizing microfluidics in the cultivation of cancer cell spheroids to develop personalized treatments for cancer [82], and more recently in ovarian cancer [83]. However, limited information is available about the use of microfluidics for human ovarian cortex culture to grow primordial follicles to antral stages to produce mature oocytes, especially useful for fertility preservation in pre-pubertal girls. Promising results have been published for follicular culture using a OOC platform in large mammals. A microfluidic device combining alginate and collagen to fabricate cortical and medullar tissue in the ovary to culture secondary follicles from deer mice has been studied [70]. Nagashima et al. [84] reported the effect of flow on the culture of preantral follicles of dogs and cats. In a recent study, Xiao et al. [85] created the microfluidic platform EVATAR simulating the in vivo female reproductive tract to replicate the 28-day menstrual cycle. One of the main advantages of this technology is the use of human samples (except the ovaries that were obtained from rats), to help to understand diseases in the female tract.

**Figure 5:**
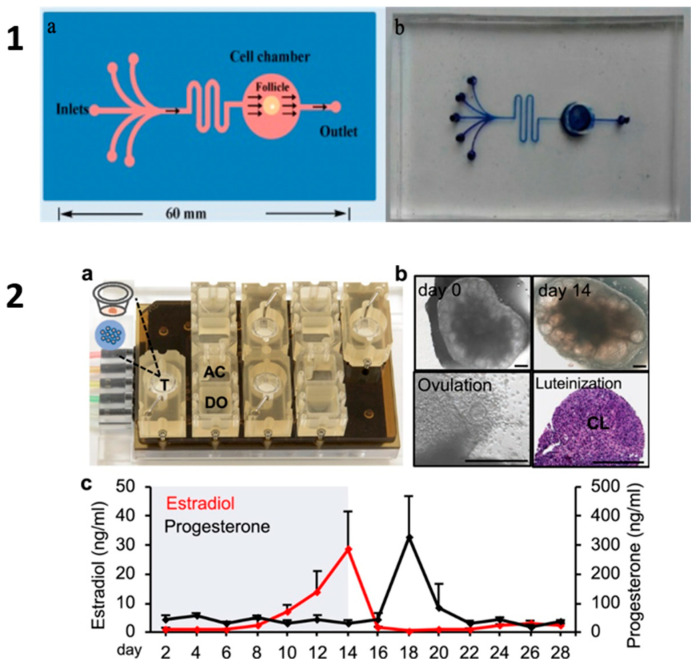
Ovary-on-a-chip. (**1**) A PDMS microfluidic device designed for ovarian follicle culture by Aziz et al. [86]. (**a**) Schematic representation of the device featuring 5 inlets and 1 outlet, containing a single chamber for follicle cultivation. (**b**) Real microfluidic device. (**2**) A microfluidic platform for culturing individual follicles and ovarian explants developed by Zubizarreta and Xiao [81]. (**a**) Each microfluidic platform comprises 4 replicates of a fluidic circuit, with each replicate containing three interconnected modules: the inlet media donor module (DO), tissue culture module (T), and outlet media acceptor module (AC). (**b**) The ovary-on-a-chip supported the extended culture of mouse ovarian explants, facilitating follicle development, oocyte maturation, ovulation, and luteinization. CL: corpus luteum. Scale bar: 300 μm. (**c**) The ovary-on-a-chip generated a 28-day menstrual cycle-like hormone secretion profile, encompassing both the follicular and luteal phases.

However, as we are aware of the limitations of the murine model for the development of in vitro folliculogenesis for other species, there is still no evidence that a dynamic culture system succeeds in the follicular growth from girls or woman in vitro. Among the human studies, a microfluidic chip was used to culture hydrogel human pre-antral follicles encapsulated in alginate, confirming the successful growth of ovarian follicles with their hormonal trends and diameter increase [86] (OOC from Aziz et al. [86] and Zubizarreta and Xiao [81] are illustrated in Figure 5).

Park et al. [87] developed a novel dual reproductive Organ-on-a-Chip between the uterine endometrium and the ovary that reflected the bidirectional endocrine cross-talk between the two tissues through media sharing between the channels, improving the viability of the loaded cells. However, the use of a microfluidic approach for the purpose of maturing human ovarian follicles has not been well studied or applied. Recently, Sood et al. [88] reviewed the use of microfluidics in human reproductive organs, emphasizing the challenges for the clinical and scale-up dynamics of these technologies. Future studies should investigate long-term culture and identify the optimal flow rates. Microfluidics could potentially provide a powerful tool to preserve fertility, providing the 3D environment necessary to maintain architecture over a long culture period, adequate nutrition and oxygenation, and permitting the sequestration of autocrine/paracrine factors within the vicinity of the follicle.

## 5. Conclusions

Ovarian follicular growth has great potential to restore fertility in young women suffering from cancer or adult women that need an imminent treatment and cannot undergo the cryopreservation of oocytes and embryos, avoiding the transplant and the risk of reintroducing malignant cells. However, the development of an optimal culture system to resemble in vivo folliculogenesis is necessary. The use of a dynamic culture system based on microfluidics, the definition of the mechanical characteristics of the matrix, and a stage-dependent modulation of this matrix composition should be addressed in order to obtain meiotically competent oocytes.

Advanced biomimetic devices such as microfluidic technology combined with ERC matrices may be valuable as a better in vitro culture system to preserve fertility, mimicking the dynamic supply of substances and gases in the ovary and recapitulating the 3D mechanical, physiological, and anatomical milieu in the ovary. This in vitro culture system is an ambitious pathway and is still maturing but may lead to a new assisted reproductive technique for clinical practice. OOC technology could revolutionize the field of reproductive biology.
